# SigsPack, a package for cancer mutational signatures

**DOI:** 10.1186/s12859-019-3043-7

**Published:** 2019-09-02

**Authors:** Franziska Schumann, Eric Blanc, Clemens Messerschmidt, Thomas Blankenstein, Antonia Busse, Dieter Beule

**Affiliations:** 1 Core Unit Bioinformatics, Berlin Institute of Health, Charitéplatz 1, Berlin, 10117 Germany; 2 Max Delbrück Center for Molecular Medicine in the Helmholtz Association (MDC), Robert-Rössle-Str. 10, Berlin, 13092 Germany; 3 Charité - Universitätsmedizin Berlin, corporate member of Freie Universität Berlin, Humboldt-Universität zu Berlin, and Berlin Institute of Health, Charitéplatz 1, Berlin, 10117 Germany; 4 Insitute of Immunology, Charité - Universitätsmedizin Berlin, corporate member of Freie Universität Berlin, Humboldt-Universität zu Berlin, and Berlin Institute of Health, Charitéplatz 1, Berlin, 10117 Germany; 5 Berlin Institute of Health, Charitéplatz 1, Berlin, 10117 Germany

**Keywords:** Cancer, Mutational signatures, Bioconductor

## Abstract

**Background:**

Mutational signatures are specific patterns of somatic mutations introduced into the genome by oncogenic processes. Several mutational signatures have been identified and quantified from multiple cancer studies, and some of them have been linked to known oncogenic processes. Identification of the processes contributing to mutations observed in a sample is potentially informative to understand the cancer etiology.

**Results:**

We present here SigsPack, a Bioconductor package to estimate a sample’s exposure to mutational processes described by a set of mutational signatures. The package also provides functions to estimate stability of these exposures, using bootstrapping. The performance of exposure and exposure stability estimations have been validated using synthetic and real data. Finally, the package provides tools to normalize the mutation frequencies with respect to the tri-nucleotide contents of the regions probed in the experiment. The importance of this effect is illustrated in an example.

**Conclusion:**

SigsPack provides a complete set of tools for individual sample exposure estimation, and for mutation catalogue & mutational signatures normalization.

**Electronic supplementary material:**

The online version of this article (10.1186/s12859-019-3043-7) contains supplementary material, which is available to authorized users.

## Background

Throughout their lives, cells are exposed to many different influences that can compromise the integrity of their DNA by introducing changes to the genome [1]. These somatic mutations are randomly introduced into the genome by various biochemical processes. These processes have different affinities for local genomic sequences, so that they leave their mark in the form of a specific mutation pattern on the genome of the cancer cell [2]. These patterns are termed (somatic) mutational signatures. Alexandrov et al. [3] have extracted and characterized a first set of mutational signatures and more signatures are being reported continuously.

The catalogue of somatic mutations in cancer, short COSMIC [4], hosts various sets of consensus signatures that have been found during analysis of the aggregation of multiple datasets from distinct types of human cancer [4, 5]. Some of these mutational signatures have been linked to environmental factors, like tobacco smoking or UV-light and the constituting DNA repair-mechanisms [3, 6], others have been associated with intrinsic processes such as defective DNA mismatch repair [7, 8]. The detection of these signatures in a tumor sample can thus yield helpful insights about the cancer’s aetiology [3] for diagnosis, prevention [9] and therapy [10].

Different frameworks have been proposed to mathematically decipher whether provided reference signatures are present in sequencing data from a single patient and how much they each contributed to its mutational load [11, 12]. Following Alexandrov et al. [3], the mutations are defined as the 6 single nucleotide variants (C >A:G >T, C >G:G >C, C >T:G >A, T >A:A >T, T >C:A >G & T >G:A >C) flanked by one nucleotide on each side. The tri-nucleotide formed by the mutated nucleotide and its two neighbours is called the *context* of the mutation. Mathematically, the mutational profile derived from sample data (*mutational catalogue*), can be expressed as vector **m** of *K*=96 somatic mutation frequencies that have been observed in the cancer sample. Mutational signatures are described by a matrix **P** which elements *P*_*kn*_ reflect the frequency with which the mutational process corresponding to the *n*^*th*^ signature causes the *k*^*th*^ mutational feature [3]. As the exposure of a mutational profile to a signature represents the signature’s contribution to the mutational load of the former, exposures to a set of processes can be inferred from a mutational catalogue by minimizing the difference between the observed & reconstructed catalogues:

$$  \mathbf{e} = \underset{e_{n} \in \mathbb{R}_{\geq 0}}{\text{argmin}} \Vert \mathbf{m} - \mathbf{P} \mathbf{e} \Vert_{2}   $$

The reconstructed catalogue **Pe** is the product between the mutational profiles matrix **P** and the exposure vector **e**, when individual exposures *e*_*n*_ are restricted to non-negative values. This formalism assumes that different processes have additive contributions to the mutational load.

We describe SigsPack, a Bioconductor package to estimate exposures to processes described by a known mutational signature matrix, for example from COSMIC. SigsPack also provides estimates of exposure stability, using bootstrapping. Its performance is benchmarked against synthetic & real data, using multiple tumor samples collected from the same patient. The effect of mutation context frequency is discussed, as well as the stability of individual COSMIC signatures and the loss of accuracy suffered by small mutational catalogues.

## Implementation

### Package description

We provide R package SigsPack for easy computation of exposures from mutational catalogues. The package provides several features, allowing to read the primary mutation data, normalize the mutational catalogues if necessary & compute the exposures with their bootstrapped variation estimates.

#### Exposure estimation

The basic functions requried to compute exposure estimates are listed below. The COSMIC signatures have been included in the package (version 2 & 3, Single Nucleotide Variants (SNV) only), and are used by default. However, it is possible for the user to import her own signature matrix, or use a sub-set of COSMIC signatures, instead of the whole matrix.

- Extract a sample’s mutational catalogue from a file in VCF format
  The function vcf2mut_cat allows to extract a mutational catalogue from a vcf file in a format so that can be used with the package (and most other packages from this field)
- Signature exposure estimation (or ’signature fitting’)
  The signature exposure is calculated using quadratic programming, in the same way as [13]. This can be done on one or several samples at once using function signature_exposure.
- Bootstrapping & variability estimation
  Following [13], SigsPack provides a function (bootstrap_mut_catalogues) to bootstrap a sample to gain a better variability estimation of the sample’s signature exposure (referred as *bootstrapping estimates*). The operation is achieved by creating multiple catalogues, each obtained by re-sampling the original catalogue with replacement. The number of re-sampled catalogues is under user control, and by default is set to 1000. That value has been used throughout the validation runs shown here.

#### Tri-nucleotide contexts & normalization

SigsPack provides several functions which allow the user to put any mutational catalogue on a scale compatible for their choice of signature matrix. These functions can also be used to perform the inverse operation, i.e. to re-scale one or more signatures to match the frequencies on which the data have been collected. Normalization is required to correct differences in tri-nucleotide context frequencies of the catalogues and the signature matrix, typically from exome and whole genome respectively.

- Extracting trinucleotide context frequencies from genomes or exomes
  get_context_freq computes the trinucleotide distribution of exomes and/or genomes, which is needed to normalize the data or signatures.
- Normalization
  normalize can be called to normalize the data to fit the signatures or vice versa. This requires the user to provide the trinucleotide frequencies of the data’s reference genome or exome (SigsPack’s function get_context_freq can extract these frequencies from an exome bed file or a BSgenome entity). The same has to be provided for the signatures in case the user chooses to provide their own signatures.

#### Other tasks

The package also provides convenience functions to visualise the results, and to generate synthetic data that can be used to analyse signatures stability.

- Plots
  Given a mutational catalogue, the function summarize_exposures bootstraps it and provides a table and a plot illustrating the results of the signature estimation for this sample and the bootstrapped re-samples. The plot shows the distribution of estimated signature exposure for all the re-samples, highlighting the one of the original mutational catalogue, thus providing insights on the reliability of the estimates.
- Data simulator
  The create_mut_catalogues function allows to create mutational catalogues with exposure to specified signatures by sampling mutations from a distribution of those signatures’ weighted profiles. The signatures can either be known consensus signatures from COSMIC (whose signature profiles are included in the package for convenience) or signatures provided by the user. These can be specified on any kind of features so that the application is not limited to the 96 mutational contexts but can also be used, for example, to simulate profiles with strand bias.

### Synthetic datasets

We used our package to construct two datasets consisting of simulated mutational catalogues using the function *create_mut_catalogues*. Samples in *set1* are drawn from a distribution formed with equal contributions of signatures 7, 13, 21, 24 and 28 (so with each a weight of 0.2), likewise, the mutational catalogues of *set2* have been samples from a distribution of signatures 3, 5, 8, 16 and 25 each having a weight of 0.2. The number of mutations in the catalogues were set to 1000, except when assessing the catalogue’s size effect on reconstructed exposures accuracy (see Fig. 3).

### Exome datasets

To quantify stability of exposure estimation with respect to biological variability, we have taken 13 different tumor samples from 3 colorectal cancer patients (4 for patients 69 & 99, and 5 for patient 80. For each patient, one single blood sample was used as normal. For all samples, the Agilent SureSelect XT Human All Exon V4 exome enrichment kit was used. BWA-mem v0.7.12 [14] was used to align each whole-exome sample against genome reference GRCh37, separate read groups were assigned for all reads from one lane, and duplicates were masked using Samblaster v0.1.24 [15]. Single nucleotide variants were called with MuTect [16] in the default configuration. The number of somatic SNVs identified in the samples ranged between 481 and 756. Mutational catalogues were normalized to the genome sequence content before computing exposures. For the quantification of the tri-nucleotide frequencies effect, exomes mutational catalogues were left un-normalized, and instead the COSMIC mutational signature matrix was re-scaled. A detailed description of normalization operations is found in Additional file 1.

## Results

### Similarity between signatures

**Fig. 1:**
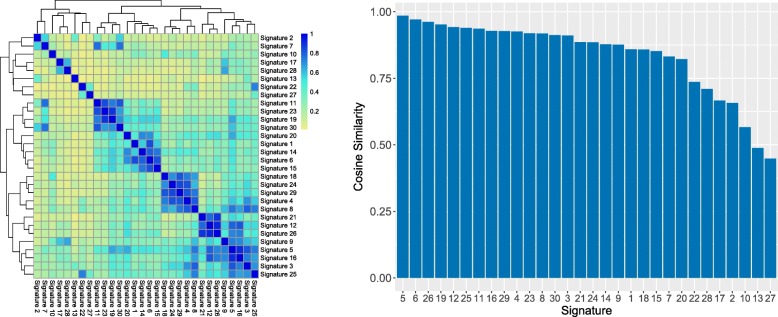
Similarity between COSMIC signatures. **a** Pairwise cosine similarity of the 30 COSMIC signatures. Co-linear signatures have a similarity of 1, and orthogonal signatures a similarity of 0. Signatures 5 & 16 have a similarity higher than 0.9. **b** Computed cosine similarity of each signature with the profile that was constructed by trying to reconstruct each signature with the 29 others. The signatures are ordered by decreasing similarity to their reconstructed profile

By construction, COSMIC signatures are non-orthogonal, in the sense that all the 96 mutation contexts are found in more than one signature. This non-orthogonality can affect the stability of the exposures. Figure 1 illustrates different aspects of the non-orthogonality between COSMIC signatures. Figure 1a displays the distance between signatures expressed as pairwise cosine similarity. Non-orthogonality effects can be assessed by computing the error between each signature profile and its reconstruction using all the 29 other signatures. Figure 1b shows that four signatures (5, 6, 19 and 26) have a cosine similarity higher than 0.95 with their reconstructed profile. Those signatures might be labelled as *unstable*, as the information contained in their profile is mostly contained in the remaining 29 others.

#### Accuracy of exposure estimation

**Fig. 2:**
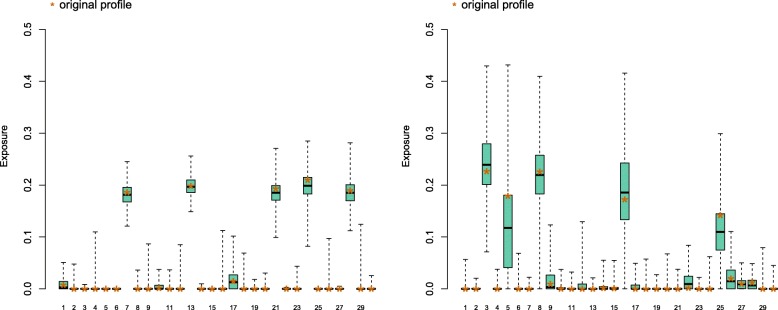
Signature prediction on a simulated mutational catalogue. The catalogue is sampled from *set1* signatures (7, 13, 21, 24 and 27), each occurring with a probability of 20% (**a**), and from *set2* (3, 5, 8, 16 and 25), with the same 20% occurring probability (**b**). In both case, the mutation catalogue consists of 1000 mutations and was bootstrapped 1000 times. The signature contribution was predicted with quadratic programming, for each re-sample the distribution is shown. The stars mark the prediction for the original profile

To investigate the possible effects of the non-orthogonality between COSMIC signatures, we have measured the accuracy of exposure reconstruction using synthetic datasets (see the “Implementation” section). Based on Fig. 1, we have selected 5 fairly distant signatures (*set1*, signatures 7, 13, 21, 24 & 27), and 5 relatively similar signatures (*set2*, signatures 3, 5, 8, 16 & 25), and generated mutational catalogues from each of them. Figure 2a shows that in certain cases, exposures can be faithfully recapitulated, and that exposures are relatively robust to small changes in the mutational catalogue. Inferred exposures for signatures absent from *set1* are very small (Additional file 2): the 3rd quartile of the bootstrapped values for those signatures is above 1% only for signatures 1 & 17, and the bootrapped maximum values above 10% for signatures 4, 16, 17 & 29. As signature estimations cannot be negative by construction, the overall effect of estimation errors is to contribute to a small underestimation of the contribution of 4 out of 5 present signatures (7, 13, 21 & 27).

**Fig. 3:**
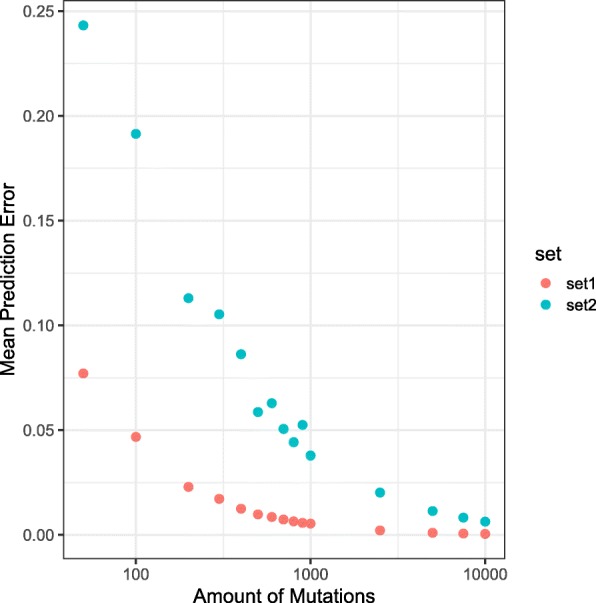
Comparison of the mean prediction error with regard to the amount of mutations in the profile. Simulated samples have been created from *set1* & *set2* with different number of mutations. All have been bootstrapped 1000 times. The plot shows the mean prediction error, i.e. the mean for all SSE between the original exposure matrix and the predicted one, as a function of the mutational catalogue size (shown on logarithmic scale)

The reconstruction of exposures in mutational catalogues drawn from *set2* (Fig. 2b & Additional file 2) shows that any small amount of noise in the data leads to dramatic changes in the exposure predictions. Exposure to signature 5 is underestimated in more than 75% of the re-sampled catalogues, and half re-sampled catalogues underestimate signature 25 contribution by almost 50%. Signature 26 (which is absent from the generation protocol) is assigned a contribution higher than 3.6% in 25% of the re-sampled catalogues.

#### Required number of mutations

In order to quantify how the precision of the inferred exposures depends on the mutational load of a sample, simulated mutational catalogues based on *set1* and *set2* have been created with different amounts of mutations. Figure 3 displays the mean prediction error, i.e. the mean difference between the predicted signatures exposure and the actual one. In general, we observe that the mean prediction error is higher on samples with only a small mutational load, consistent with Rosenthal et al. [12]. We also notice that the prediction error is lower on *set1*, constructed with *stable* signatures, than it is on *set2*, constructed with *unstable* signatures, even for high mutational loads.

#### Bootstrapping can faithfully recover the biological variance

**Fig. 4:**
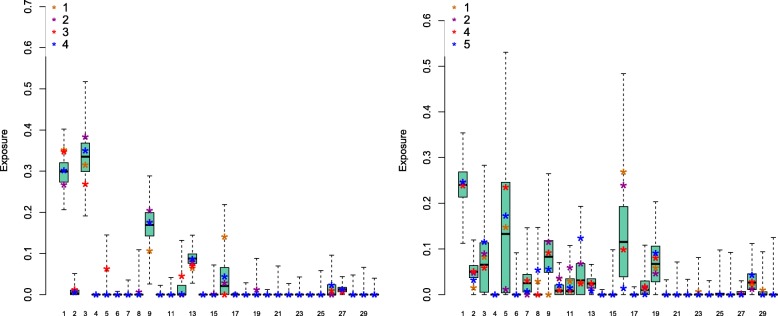
Exposures estimations for multiple samples from the same patient. **a** Patient 99, variability intervals obtained by bootstrapping sample 4. **b** Patient 69, variability intervals obtained by bootstrapping sample 4. In both cases, 1000 re-sampling realisations of each catalogue have been used by bootstrapping

Next, we have investigated the stability of exposures inference in the presence of biological variability, using the 13 samples described in the “Implementation” section. As the experimental protocol was identical for all samples, the differences between inferred inferences within the same patient could then be attributed mostly to the heterogeneities in the clonal compositions of each sample. Figure 4 shows two examples of the agreement between exposure reconstruction from different samples. In that figure, the bootstrapping estimates have been computed from a single sample (the complete set of plots can be found in Additional file 7).

If we postulate that boostrapping can provide estimates to exposure variability, then the bootstrapping intervals computed from the different samples should largely overlap, and the overlap should contain the original exposures’ estimation values. To test this hypothesis, we have computed, for each patient and each signature, the intersection of the exposure range obtained by bootstrapping from all samples. We have then asked whether any exposure estimated for individual samples fell outside of this range. This happens only for signature 27 (patients 69 & 80) and 5 (patient 99). In two out of three cases, the exposure estimation is higher than 0 (0.2% & 6%) for one sample, while the range is limited to 0 for another sample. In the third case, (signature 27 in patient 80), the exposure is estimated to be 2.49% in sample 2, while the bootstrapping range computed from sample 3 is between 0 and 2.43%. We conclude from these examples that in most cases, the boostrapping variability estimates can provide a proxy for the exposures’ variability due to clonal heterogeneity of mutational catalogues.

Across all patients, the contribution from unstable signatures vary considerably from one sample to the next, and there is more than 10% difference between estimates from the 4 samples for signatures 5, 9 & 16 for patient 69, and for signatures 3 & 16 for patient 99 (the corresponding signatures for patient 80 are 3, 5, 8 & 16). As for synthetic data, bootstrapping provides large variation estimate for those signatures: for signatures 5 & 16, the extreme values reached by bootstrapping differ by more than 30% for 10 out of the 13 samples. These difference appear to be compensating: across the 13 samples, the correlation between signatures 3 & 16 exposures is −0.75, the most negative correlation between all signature pairs. This observation supports the hypothesis that the unstable mutations can be explained almost equally well by their contributions. The complete set of exposures is found in Additional file 4. Signatures 6 & 10, often observed in colorectal cancers, are not present at high levels in any of the 13 samples analysed here. However, these signatures are associated with microsatellite instability (MSI) and mutations in POLE and the DNA repair mechanism [17]. Neither MSI nor POLE mutations were present in any of the 3 patients.

#### An example of the sequence composition effect

**Fig. 5:**
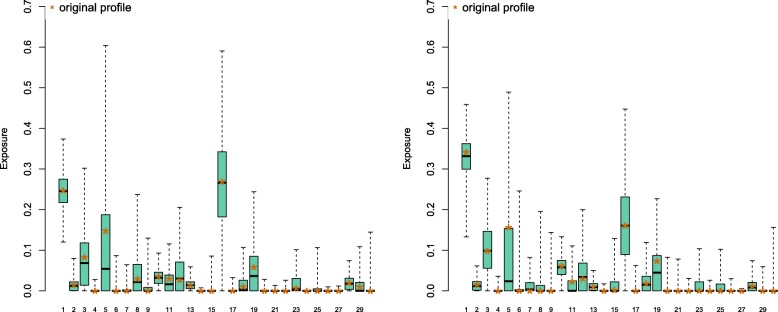
Exposures estimations for different normalization of the same sample. **a** Normalization on the genome, where the observed mutation frequencies were re-scaled. To perform the scaling, tri-nucleotide ratios where first obtained by dividing the tri-nucleotide frequencies observed in exome regions by their corresponding values in the whole genome. The mutational catalogue was then scaled by the tri-nucleotide ratio and converted to frequencies. **b** Normalization on the exome, where the COSMIC signatures were re-scaled. In this case, the COSMIC signatures were scaled by the inverse of the tri-nucleotide ratios, and converted to frequencies again. Together, **a**) & **b**) show that the scaling direction (mutational catalogue or signatures) lead to different exposures estimations. In both cases, 1000 realisations have been used by bootstrapping

COSMIC signatures have been normalized to the trinucleotide frequencies of the human reference genome version GRCh37 [18]. The regions on which the mutations catalogues have been observed directly affect the exposures estimation (see Additional file 1 for details). In particular, in presence of mutational catalogues generated from exome data, the scaling of the mutational catalogue to the genome tri-nucelotide frequencies in not equivalent to the scaling of mutational signatures to the exome tri-nucleotide frequencies. Figure 5 illustrates the sequence composition effect for the first sample of patient 69 (the full set of exposure estimations after both normalization are found in Additional file 6). Although the frequencies of enriched regions and of the whole genome are reasonably similar (correlation coefficient above 0.72, Additional file 5), there is a difference greater than 10% in the inferred exposures of signature 16. While this might be attributed to the unstability of signature 16, signature 1 also shows a difference of 9.4% in exposure estimation, due to normalization. Over all samples and all patients, signature 1 appears to be most sensitive to the scaling choice: the estimation differs by more than 10% in 11 of the 13 samples, even more often than unstable signatures (signature 16 estimation is affected in 4 samples). As signature 1 is associated with endogenous C to T mutations, its contribution is mainly affected by the tri-nucleotides containing a central C. These are more frequent (relatively) in the exome regions than in the whole genome (Wilcoxon test *P* value 4.7·10^−4^). This simple example shows that taking sequence composition into consideration by normalisation of mutational catalogues is required to ensure accurate exposure values.

#### Exposures from random catalogues

**Fig. 6:**
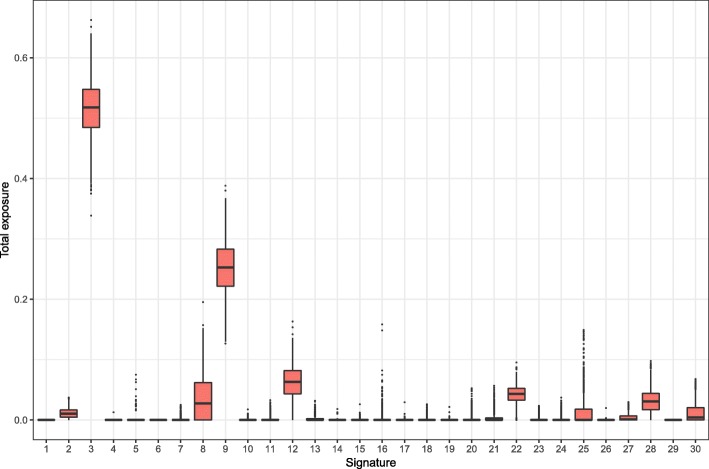
Estimated exposures from 1000 random mutational catalogues. These catalogues have been randomly drawn according to the human genome tri-nucleotide frequencies. Each catalogue has 1000 mutations

To gain understanding on possible causes of the unstability of estimation of some signatures, we have created 1000 mutational catalogues of 1000 events each, drawn with probability proportional to the underlying tri-nucleotide frequencies in the human genome. Exposures were computed from these mutational catalogues, and the sum of estimated exposures over the 1000 samples is shown in Fig. 6. These "null" catalogues represent the absence of mutational process signal, as the occurrence frequencies reflect the corresponding frequencies in the genome. Estimated exposures from signatures 3 & 9 are higher than 50% and 25% respectively for more than half of the catalogues. However, 5 or 16 are consistently absent of the exposure estimation from most "null" catalogues, unlike the unstability displayed in presence of signal. In this respect, the lack of stability in the exposure estimation cannot be solely attributed to the truly random component of the mutational catalogue, which frequency approximates the tri-nucleotides frequencies in the genome.

## Discussion

Using both synthetic data & multiple samples from the same donors, we have shown that stability of exposure estimates can be accurately represented by bootstrapping mutational catalogues. It should be noted that reliable exposure estimation can only be achieved for a relatively large mutational catalogue. When the number of available mutations falls below a few hundreds, the number of observations in each of the 96 mutation classes is not sufficient to ensure a stable exposure reconstruction. SigsPack will warn the user when the mutational catalogue size is below 125.

Even for larger mutational catalogues, some exposures displayed considerable variability upon re-sampling of the mutational catalogue. Inspection of relationships between signatures suggest that similarity between signatures, and the fact that some signatures can be approximated by others, may cause unwanted sensitivity to mutational catalogue details for some signatures’ exposures.

To overcome the problem of unstable signatures, it may be possible to follow for example Letouzé and co-workers [19] and use only a subset of signatures known to be involved in a specific cancer entity. Careful selection of signatures would presumably reduce considerably the redundancy shown in the COSMIC set, and decrease the exposures variability. The signature selection might also be guided by the particulars of the cohort under consideration. Also, for some mutational processes, the analysis of the presence of specific di-nucleotide mutations and/or indel in the patient’s somatic mutations might be more informative than the quantification of the associated signatures [10]. Such signatures might possibly be omitted, again leading to a decrease of exposures variability.

We have shown that accurate exposures estimation requires matching tri-nucleotide frequencies between regions on which mutational catalogues and signature matrix have been collected. When using the COSMIC signature matrix provided with SigsPack, exome data must be put on “genomic scale” prior to exposures estimation. Choice of reference tri-nucleotide frequencies and normalization should be carefully selected when creating new mutational signature matrix from a large cohort. Even though SigsPack doesn’t provide algorithms to generate such matrix, it allows for easy normalization of each of its component. We suggest that scaling observations to the whole genome’s tri-nucleotide frequencies should be encouraged, as it renders the results independent of the experimental particulars.

The analysis above has been carried out on the full set of COSMIC version 2 signatures. However, as the recently released version 3 contains more signatures, the signatures will remain non-orthogonal, and there might be cluster of signatures very similar to each other. These features could presumably lead to the same stability problems for exposure estimation as with COSMIC version 2 signatures.

## Conclusions

Many computational methods aimed at mutational signatures discovery already exist [20]. The SigsPack package is aimed at estimating exposure to known mutational signatures, rather than the process of uncovering new ones. It relies on the Alexandrov additive mutational frequency model, rather than a position weight matrix [21] or a probablistic framework, such as EMu [22] or sigfit [23]. It builds on existing methods ([11–13]), and provides support for vcf input, exome regions normalization, exposure estimation and stability estimates for individual signatures. It also provides facilities to plot and examine estimated exposures, and a data generation module to benchmark user’s defined signature matrices.

## Availability and requirements

- **Project name** SigsPack
- **Project home page** https://github.com/bihealth/SigsPack
- **Operating system(s)** any OS running R [24] & Bioconductor [25]
- **Programming language** R
- **Other requirements** Bioconductor
- **License** GPL-3
- **Any restriction to use by non-academics** None

## Additional files

Additional file 1 Derivation of the tri-nucelotide frequency effect on exposures. The effect of tri-nucleotide frequencies on the exposure reconstruction is detailed. (PDF 95 kb)

Additional file 2 Exposure estimation for synthetic data example. Exposure estimation from synthetic data. 1000 mutations have been generated at random, using frequencies from *set1* (signatures 7, 13, 21, 24 & 28), and *set2* (signatures 3, 5, 8, 16, & 25). A perfect reconstruction would have original exposure values very close to 0.2 for these 5 signatures, and 0 for all the others. The mutational catalogues was resampled 1000 times, and for each signature, the minimum, first quartile, median, third quartile and maximum values of exposures computed from the re-sampled data are shown. (TSV 5 kb)

Additional file 3 Mutational catalogues of the 13 samples used to estimate exposure stability in presence of biological noise. There are 4 samples for patient 69, 5 for patient 80 and 4 for patient 99. All catalogues have been collected using the Agilent SureSelect XT Human All Exon V4 exome enrichment kit. (TSV 3 kb)

Additional file 4 Exposure estimation for 13 samples from 3 colon cancer patients. Exposure estimation for 13 samples from 3 colon cancer patients (4, 5 & 4 samples from patients 69, 80 & 99 resp.). The mutational catalogues was resampled 1000 times, and for each signature, the minimum, first quartile, median, third quartile and maximum values of exposures computed from the re-sampled data are shown. (TSV 34 kb)

Additional file 5 Tri-nucleotide frequencies in the human genome & exome kit. Counts of tri-nucleotides in the human genome GRCh37 (Genome) and in the regions covered by the Agilent SureSelect XT Human All Exon V4 kit (Exome). The counts aggregate the tri-nucleotide sequence shown in the Cotext column and its reverse complement. (TSV 1 kb)

Additional file 6 Tri-nucleotide frequency effect in 13 samples from 3 colon cancer patients. Exposure estimation for 13 samples from 3 colon cancer patients (4, 5 & 4 samples from patients 69, 80 & 99 resp.), after normalization of the mutational catalogue to the genome tri-nucleotides frequencies (Genome) and after normalization of the mutation signatures to the regions enriched by the Agilent SureSelect XT Human All Exon V4 kit (Exome). (TSV 14 kb)

Additional file 7 Plots of exposure estimation for 13 samples from 3 colon cancer patients. Plots of exposures presented in Additional file 4. For each patient, the exposure variability estimation obtained from bootstrapping are plotted for all samples. (PDF 43 kb)

## Data Availability

The software package has been submitted to Bioconductor ([25]) and is available from github (https://github.com/bihealth/SigsPack). The patients mutational catalogues are included in Additional file 3. The tri-nucleotides frequencies for normalization are included in Additional file 5.

## Abbreviations

COSMIC: Catalogue of somatic mutations in cancer [4]
MSI: Micro-satellite instability
SNV: Single Nucleotide Variant
