# Supplementary material for: SigsPack, a package for cancer mutational signatures
Source: BMC Bioinformatics. 2019 Sep 2;20:450. doi: 10.1186/s12859-019-3043-7 (PMC6720940; doi:10.1186/s12859-019-3043-7)
Supplement: Supplementary file 1 — Derivation of the tri-nucelotide frequency effect on exposures. The effect of tri-nucleotide frequencies on the exposure reconstruction is detailed. (PDF 95 kb) [file 12859_2019_3043_MOESM1_ESM.pdf]

Derivation of the tri-nucelotide frequency effect on exposures

Franziska Schumann, Eric Blanc, Clemens Messerschmidt & Dieter Beule, Core Unit Bioinformatics, Berlin Institute of Health

April 16, 2019

A mutational signature essentially represents the set of 96 frequencies with which a mutation k caused by a specific process n substitutes a tri-nucleotide t_i into another tri-nucleotide T_j. Mathematically, these signatures are not defined conditionally to the original tri-nucleotide t_i (as they would sum up to 1 for each t_i), instead with respect to the tri-nucleotide frequencies in the regions captured by the original datasets (genomes or exomes). This has important implications for exposure inferrence, as the tri-nucleotide frequencies of regions covered by exome panels routinely used to collect mutational catalogues may be slightly different than the genome's.

To illustrate this fact with an example, we consider that a mutational catalogue (with frequencies $\mathbf{m}^{(E)}$) has been collected on some exome panel. The tri-nucleotide frequencies in the regions covered by the exome enrichment is slightly different than in the whole genome, and their ratio (exome to genome frequencies) are denoted by r_k. We also assume that the set of signatures $\mathbf{P}^{(G)}$ is using the genome as reference, as it is the case for the signatures provided by COSMIC. Exposures can be determined either by scaling the observed mutation rates to the genome, or by scaling the signatures to the exome trinucleotide frequencies. In the first case, the mutation frequencies $m_k^{(E)}$ are divided by r_k, to obtain an estimation of the mutation frequencies that would have been observed if data had been collected on the whole genome. In the second case, the signatures are multiplied by r_k, to correct for the difference in tri-nucleotide frequencies. We have two objective functions, one on the genome ($\phi^{(G)}$) and the other on the exome regions ($\phi^{(E)}$), and their minimisation leads to two sets of inferred exposures $\mathbf{e}^{(G)}$ and $\mathbf{e}^{(E)}$. They are defined as:

$$
\begin{aligned}
\phi^{(G)} &= \sum_k \left[\frac{m_k^{(E)}}{r_k} - \sum_n P_{kn}^{(G)} e_n^{(G)} \right]^2 \\
\phi^{(E)} &= \sum_k \left[m_k^{(E)} - \sum_n (P_{kn}^{(G)} \cdot r_k) \cdot e_n^{(E)} \right]^2 = \sum_k r_k^2 \left[\frac{m_k^{(E)}}{r_k} - \sum_n P_{kn}^{(G)} e_n^{(E)} \right]^2 .
\end{aligned}
$$

When mutational catalogues are not observed on the same regions than the signatures, exposures can then be computed using the two different formulæ above, depending on the direction of scaling (to the exome or to the genome). The solutions $\mathbf{e}^{(E)}$ and $\mathbf{e}^{(G)}$ for the minimisation of $\phi^{(E)}$ and $\phi^{(G)}$ respectively, will be in general different, as the weights of each coordinate k in the mutation space are different (1 and r_k).
