# Supplementary material for: SigsPack, a package for cancer mutational signatures
Source: BMC Bioinformatics. 2019 Sep 2;20:450. doi: 10.1186/s12859-019-3043-7 (PMC6720940; doi:10.1186/s12859-019-3043-7)
Supplement: Supplementary file 7 — Plots of exposure estimation for 13 samples from 3 colon cancer patients. Plots of exposures presented in Additional file 4. For each patient, the exposure variability estimation obtained from bootstrapping are plotted for all samples. (PDF 43 kb) [file 12859_2019_3043_MOESM7_ESM.pdf]

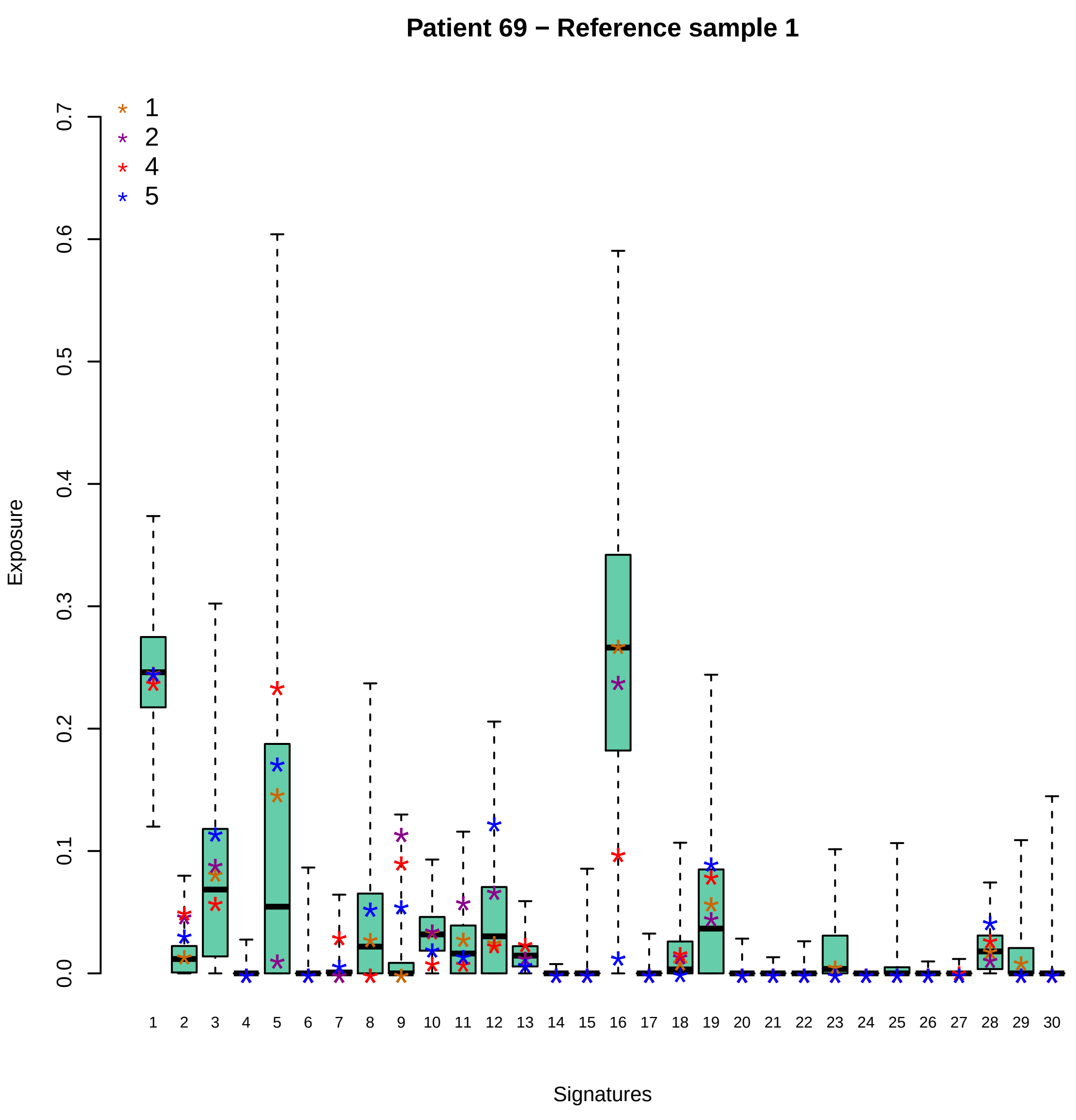
Patient 69 − Reference sample 1
1
2
4
5
Exposure
Signatures

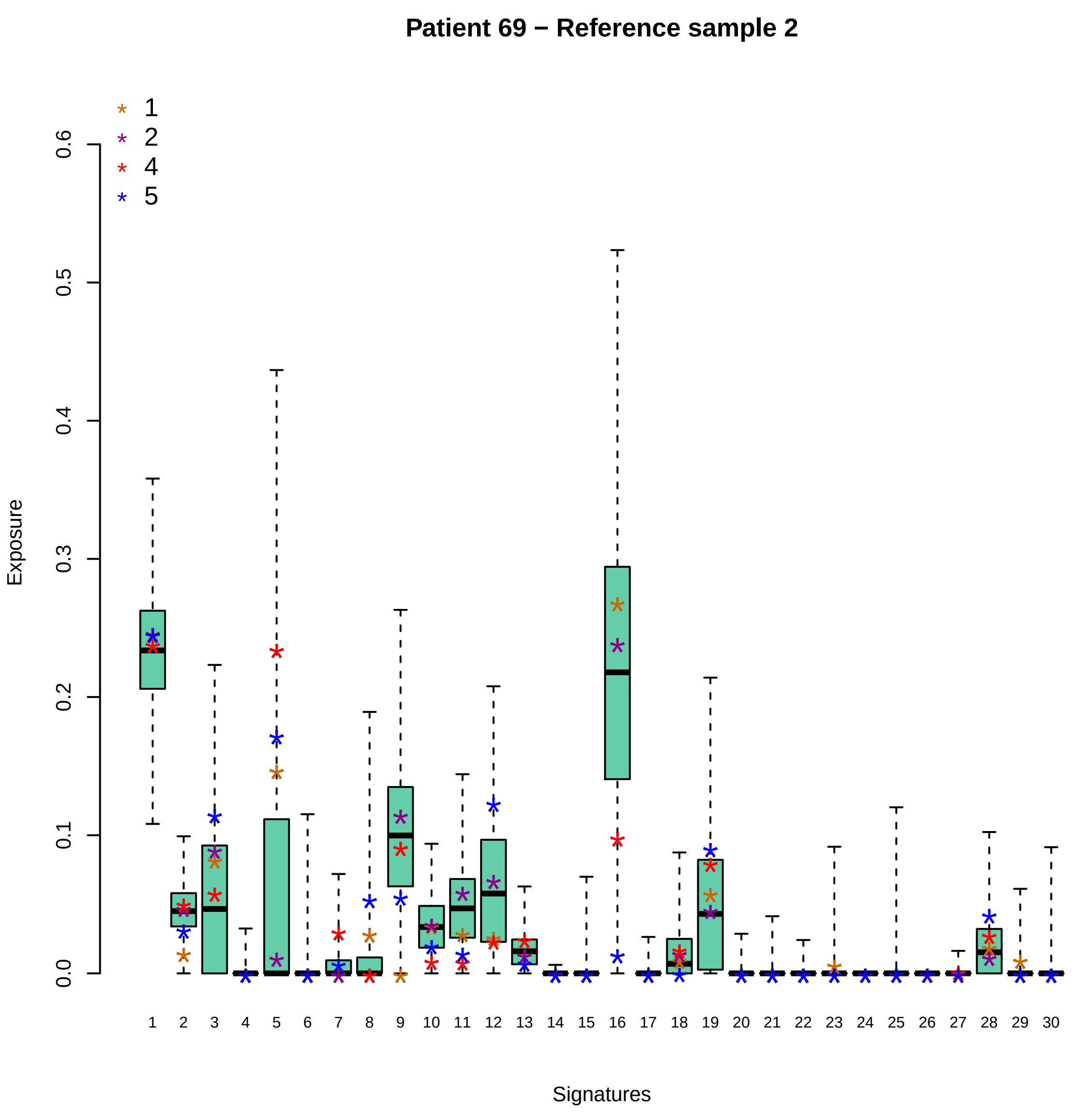
Patient 69 − Reference sample 2
1
2
4
5
Exposure
Signatures

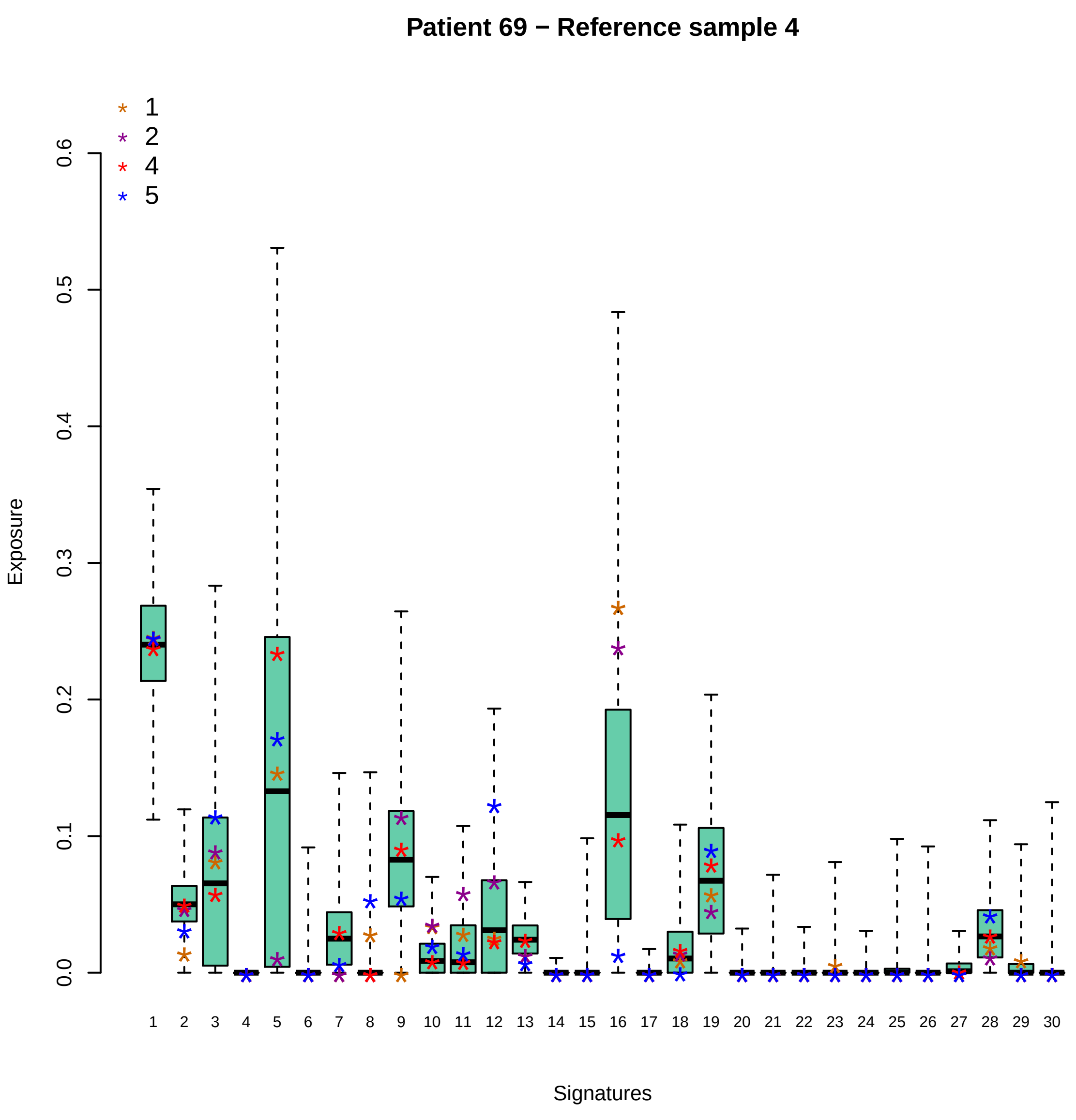
Patient 69 − Reference sample 4
1
2
4
5
Exposure
Signatures

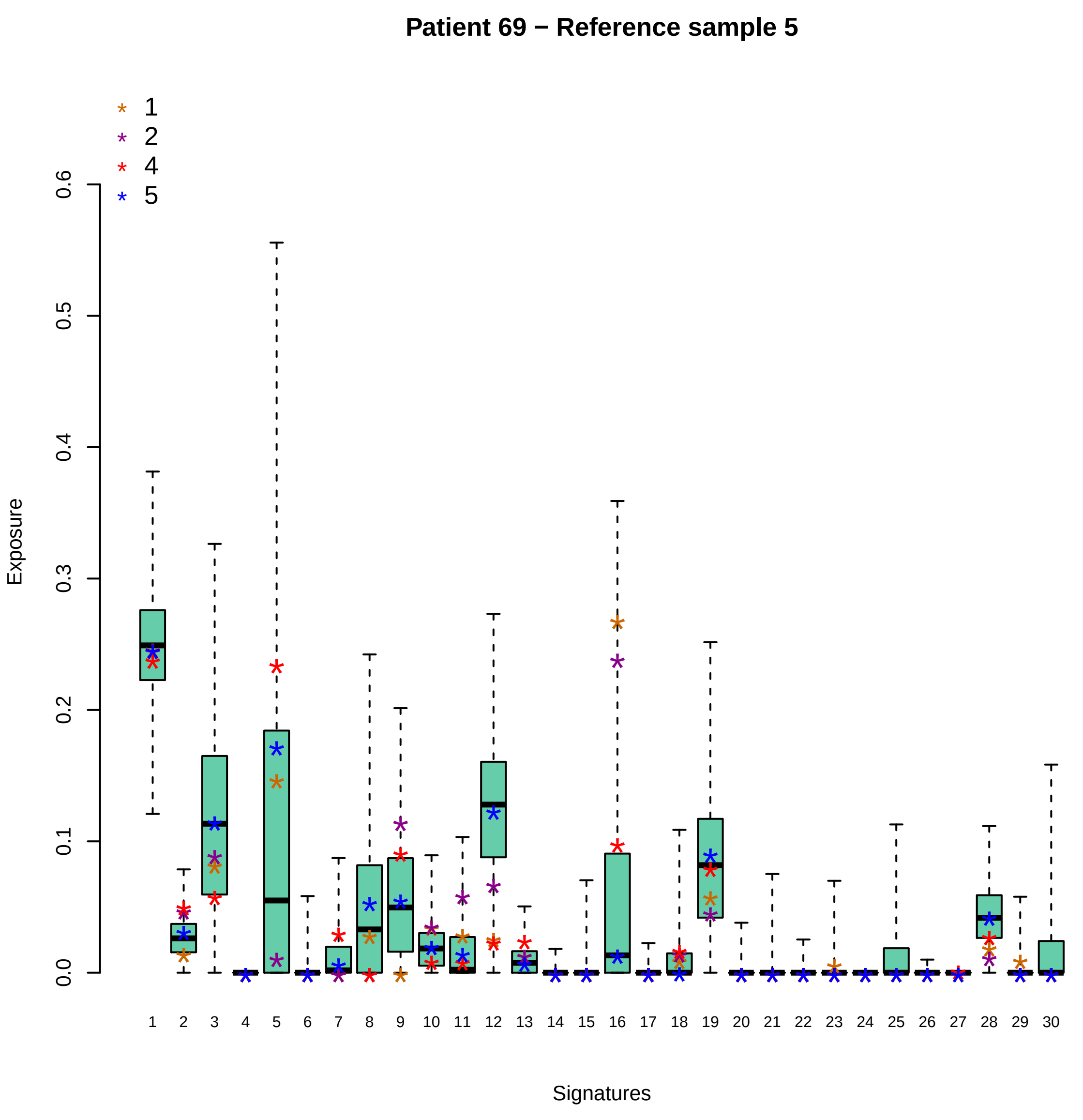
Patient 69 − Reference sample 5
1
2
4
5
Exposure
Signatures

**Patient 80 − Reference sample 1**

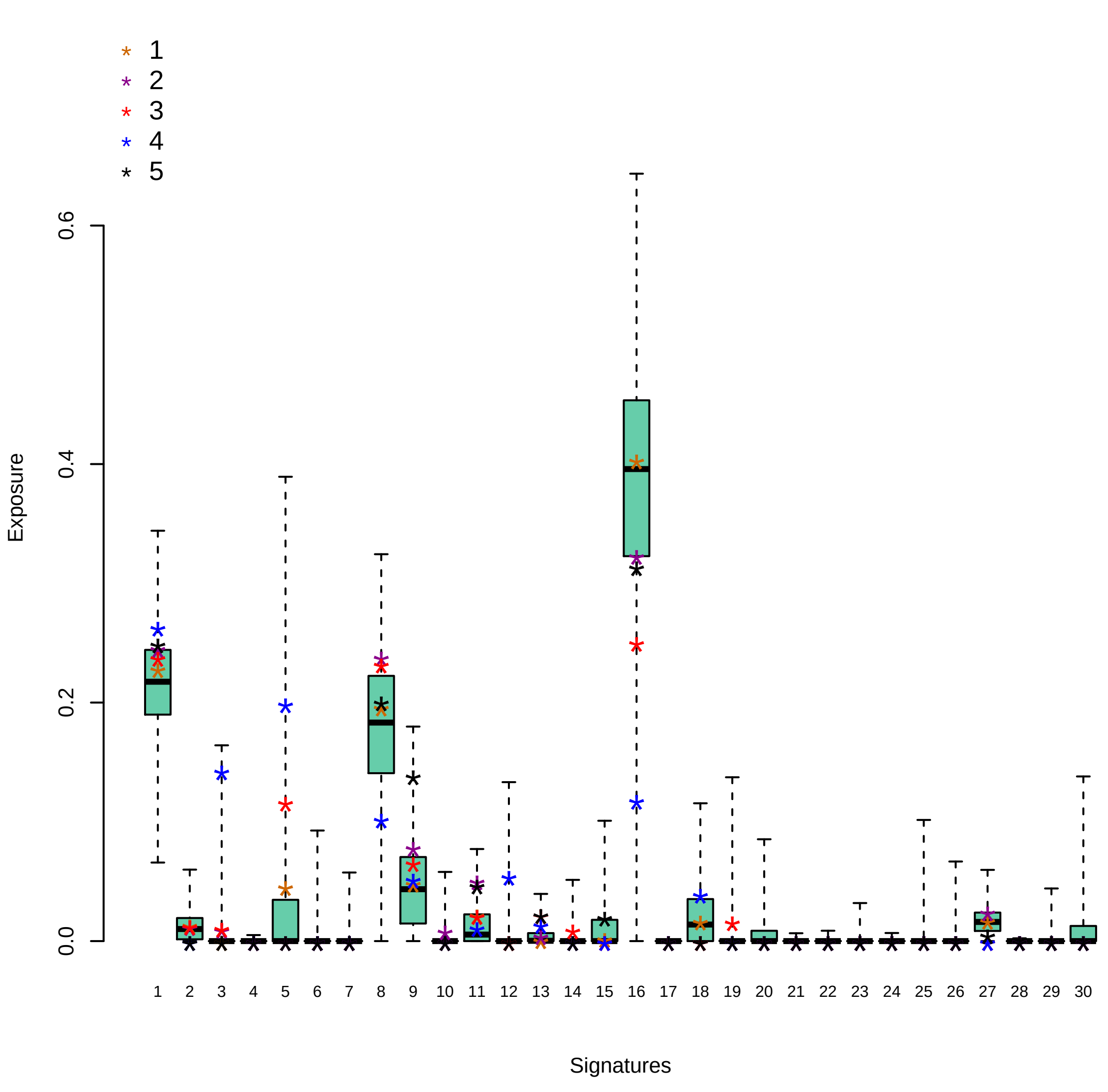


**Patient 80 − Reference sample 2**

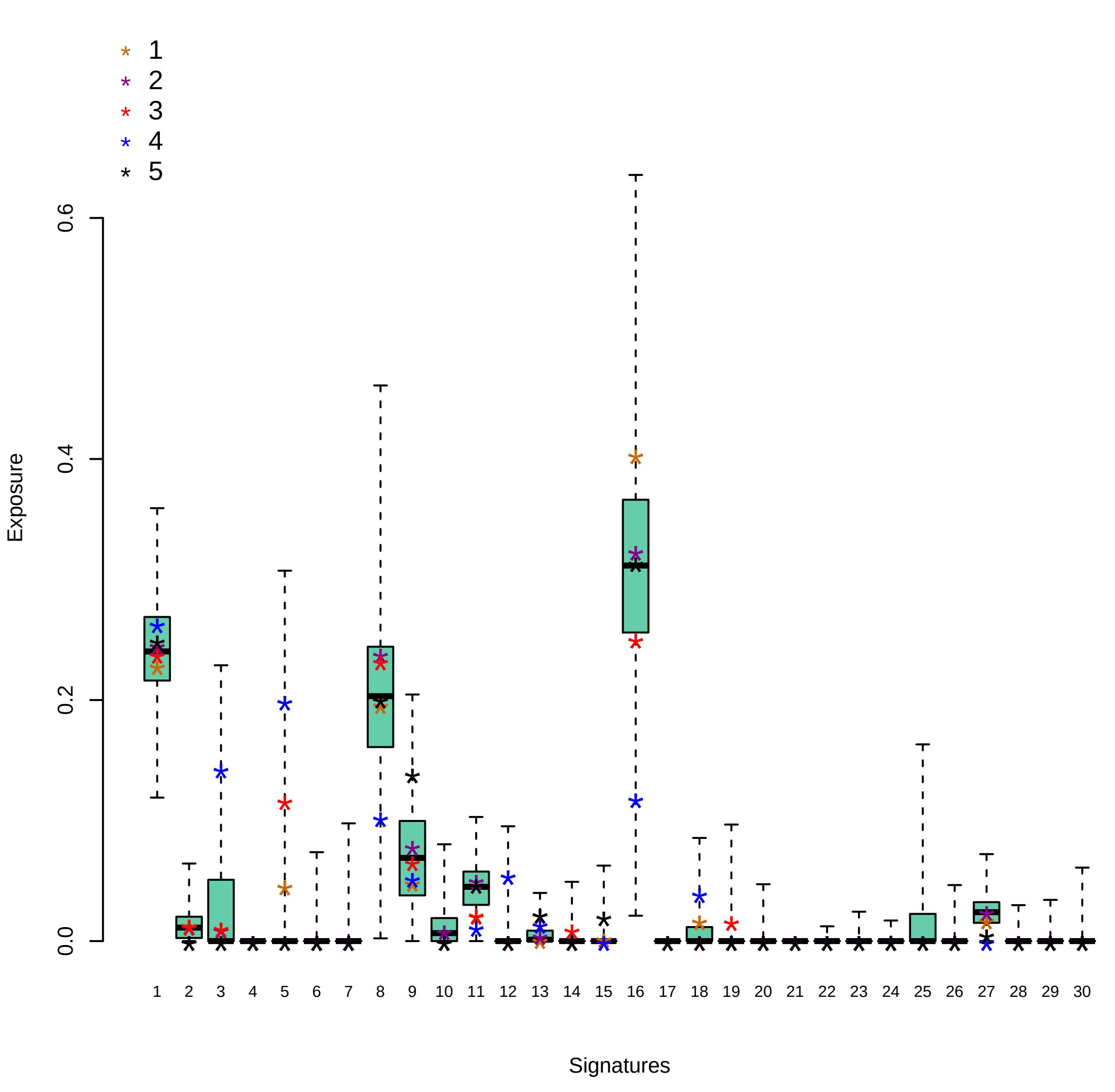


**Patient 80 − Reference sample 3**

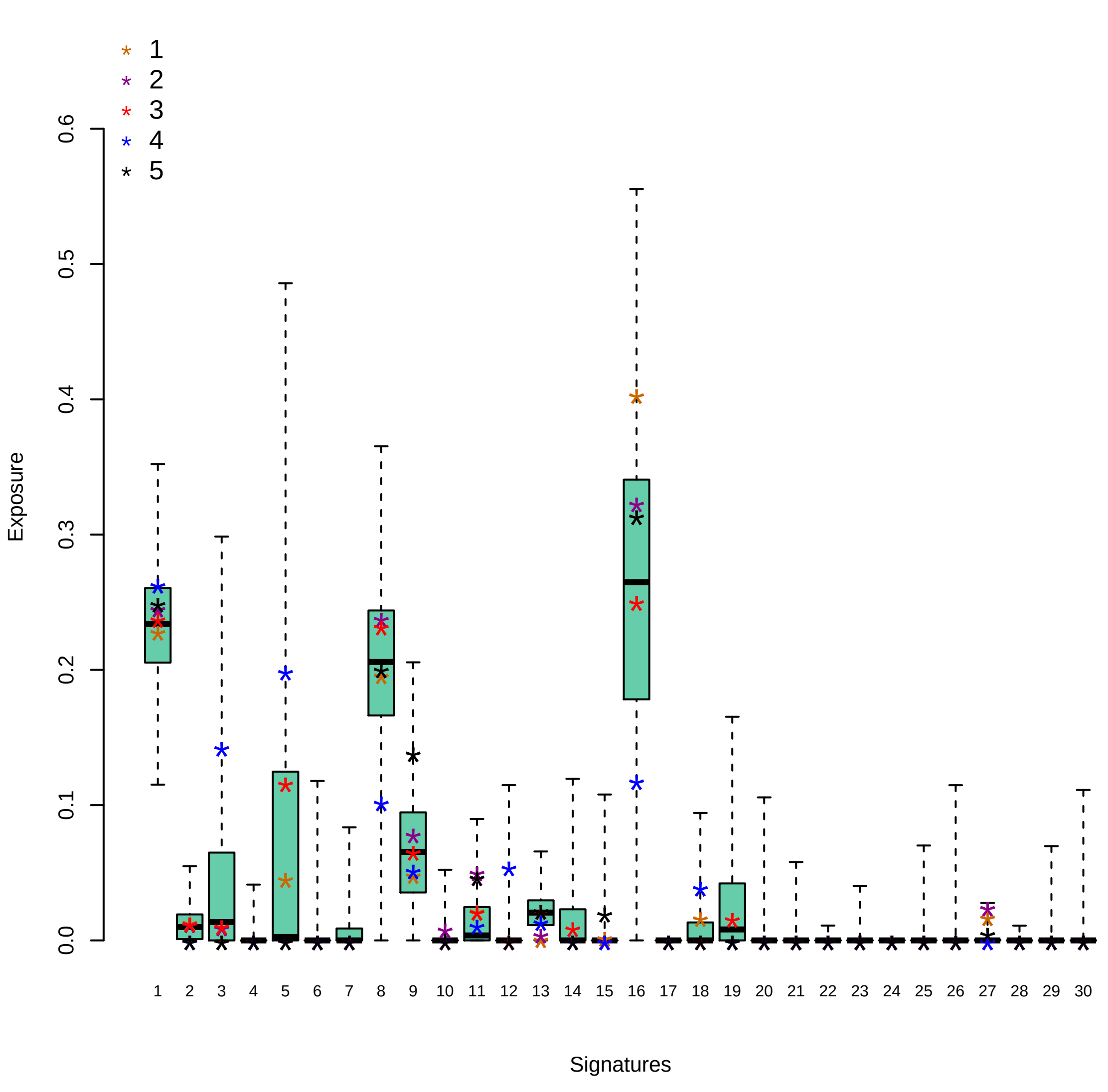


**Patient 80 − Reference sample 4**

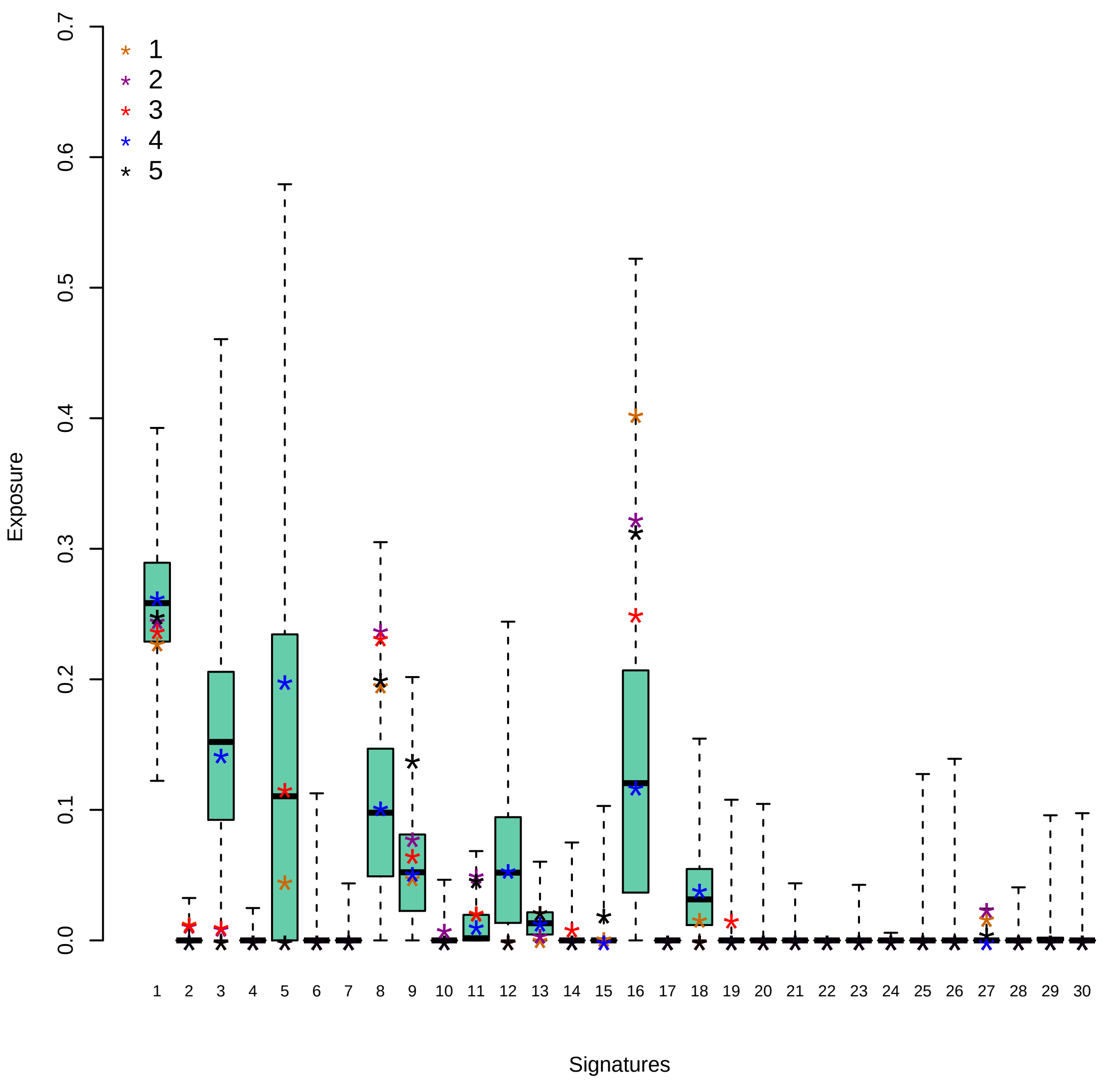


**Patient 80 − Reference sample 5**

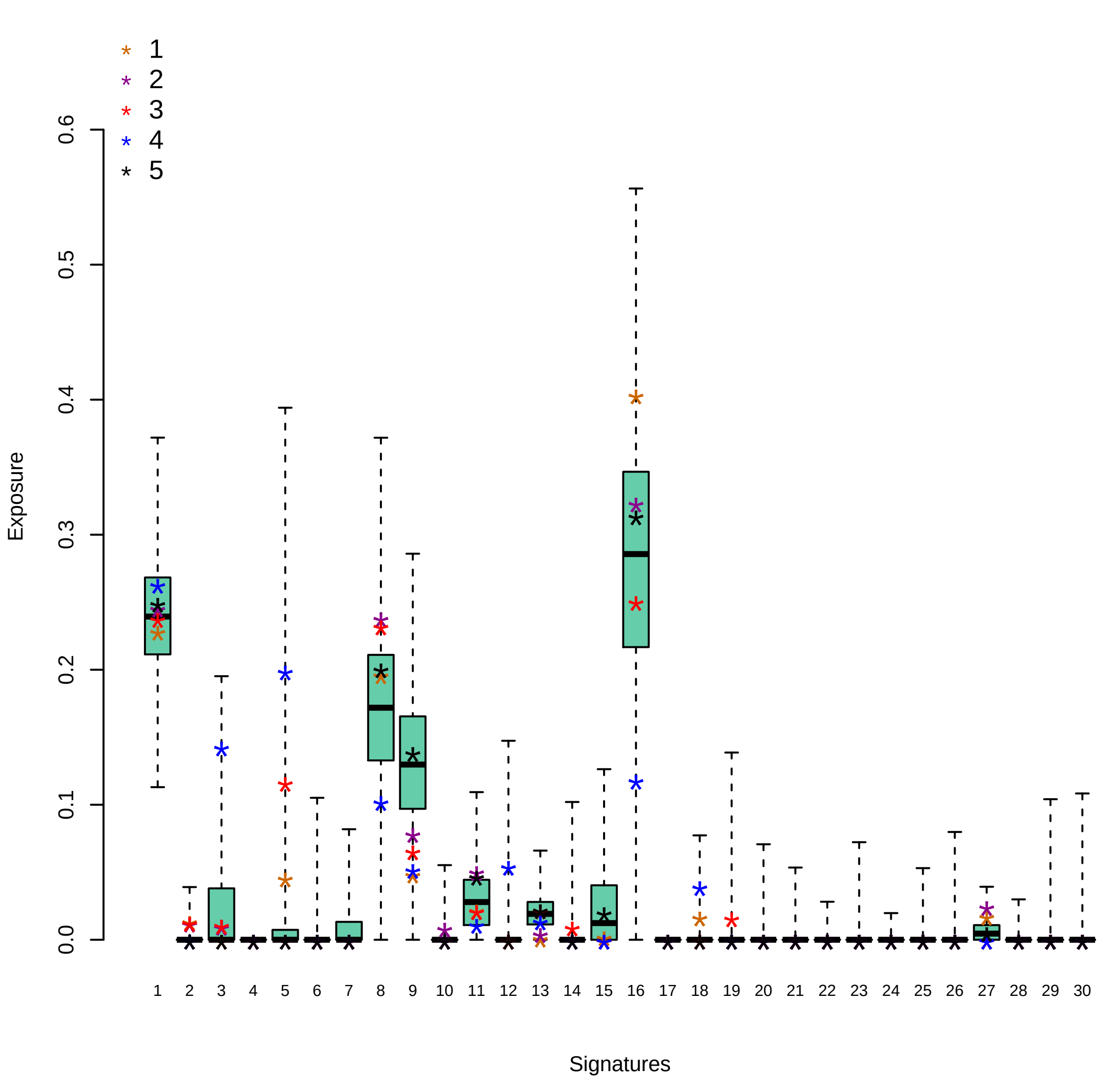

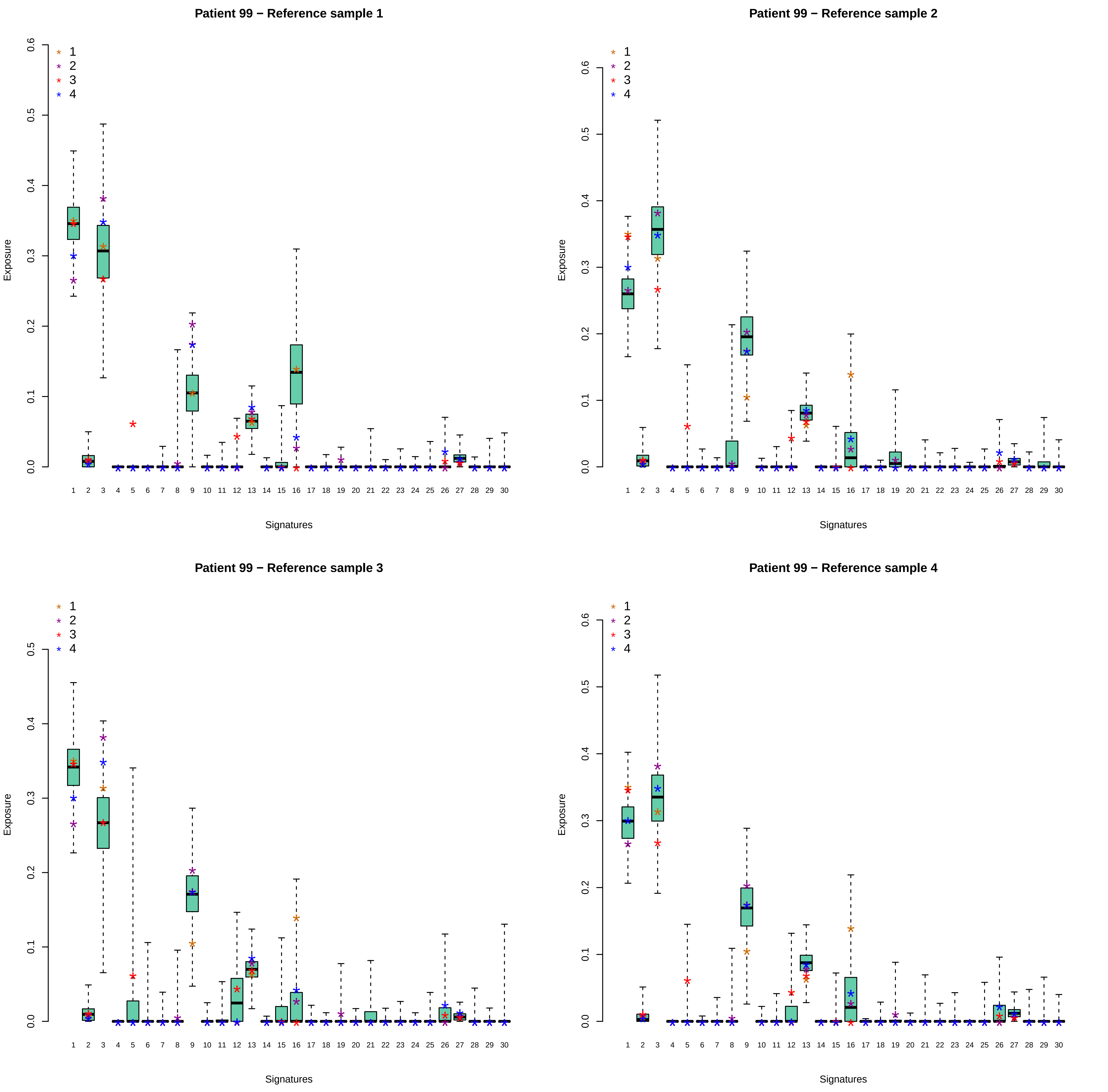

Patient 99 − Reference sample 1
Patient 99 − Reference sample 2
Patient 99 − Reference sample 3
Patient 99 − Reference sample 4
1
2
3
4
Exposure
Signatures
0.0
0.1
0.2
0.3
0.4
0.5
0.6
1 2 3 4 5 6 7 8 9 10 11 12 13 14 15 16 17 18 19 20 21 22 23 24 25 26 27 28 29 30
